# Silencing SIX1 by miR-7160 inhibits non-small cell lung cancer cell growth

**DOI:** 10.18632/aging.202398

**Published:** 2021-03-03

**Authors:** Hua-Si Zhao, Xiao-Min Tao, Qun Wang, Yuan-Yuan Fang, Hong-Yu Zhang, Hua-Qi Wang, Guo-Jun Zhang

**Affiliations:** 1 Department of Respiratory Medicine, The First Affiliated Hospital of Zhengzhou University, Zhengzhou, China; 2 Obstetrics and Gynecology Department, The Second Affiliated Hospital of Soochow University, Suzhou, China; 3 Department of Respiratory Medicine, Affiliated Zhongda Hospital of Southeast University, Nanjing, China; 4 Department of Endocrinology, Henan Provincial People’s Hospital; People’s Hospital of Zhengzhou University, Zhengzhou, China; 5 Department of Infectious Disease, The First Affiliated Hospital of Zhengzhou University, Zhengzhou, China

**Keywords:** NSCLC, miR-7160, SIX1, cancer growth

## Abstract

The homeoprotein SIX1 is upregulated in non-small cell lung cancer (NSCLC) and associated with NSCLC tumorigenesis and progression. We identified microRNA-7160 (miR-7160) as a SIX1-targeting miRNA. RNA immunoprecipitation results confirmed a direct binding between miR-7160 and *SIX1* mRNA in NSCLC cells. In the primary and established NSCLC cells, forced overexpression of miR-7160 downregulated SIX1 and inhibited cancer cell growth, proliferation, migration and invasion. Furthermore, miR-7160 overexpression induced apoptosis activation in NSCLC cells. Conversely, miR-7160 inhibition elevated SIX1 expression and enhanced NSCLC cell progression *in vitro*. Restoring SIX1 expression, by an untranslated region-depleted SIX1 expression construct, reversed miR-7160-induced anti-NSCLC cell activity. CRISPR/Cas9-inudced knockout of SIX1 mimicked miR-7160-induced actions and produced anti-NSCLC cell activity. *In vivo*, intratumoral injection of miR-7160-expressing lentivirus downregulated *SIX1* mRNA and inhibited NSCLC xenograft growth in severe combined immunodeficient mice. Significantly, miR-7160 expression is downregulated in human NSCLC tissues and is correlated with *SIX1* mRNA upregulation. Collectively, miR-7160 silenced SIX1 and inhibited NSCLC cell growth *in vitro* and *in vivo*.

## INTRODUCTION

Non-small cell lung cancer (NSCLC) is the most common type of lung cancer and a major health threat [1, 2]. In the United States alone, it is estimated that over 234,000 new cases of NSCLC will be reported each year [1, 2]. Recent improvements have been achieved for early diagnosis through emerging technologies and advanced targeted therapies. However, the five-year overall survival of NSCLC patients is still below 15%. The prognosis for the advanced NSCLC patients is extremely poor [2–6]. Considering that the incidence of this devastating disease is rising, particularly in Eastern countries [7–9], it is urgent to explore novel therapeutic targets and diagnosis biomarkers for NSCLC [10].

SIX family transcription factors have three subgroups, SIX1/SIX2 (So), SIX3/SIX6 (Optix), and SIX4/SIX5 (Dsix4) [11]. They all share a SIX-type homeodomain and SIX-domain [11, 12]. The homeoprotein SIX1 is a primary member of SIX family transcription factors. It is essential for organ development [11, 12]. SIX1 is overexpressed in human cancers and is associated with tumorigenesis [12–15]. Liu et al., have reported that SIX1 is upregulated in human NSCLC and correlated with tumor progression and poor prognosis [12].

MicroRNAs (miRNAs) are short (21-25nt long) single-stranded non-coding RNAs [16]. There are a large number of miRNAs dysregulated in human cancers [17–20]. By binding the 3′-untranslated region (3′-UTR) of targeted mRNAs, miRNAs silence gene expression post-transcriptionally and inhibits mRNA translation and/or inducing mRNA degradation [17–20]. miRNAs are involved in regulating almost all NSCLC cell behaviors. These include cell survival, proliferation, cell cycle progression, cell apoptosis and migration [21, 22]. Dysregulation miRNA has become a hallmark of NSCLC and is associated with tumorigenesis, progression and therapy-resistance [23, 24]. In the present study we identified microRNA-7160 (miR-7160) as a SIX1-targeting miRNA. Our results showed that miR-7160 expression silenced SIX1 and inhibited NSCLC cell growth *in vitro* and *in vivo*.

## RESULTS

### miR-7160 binds and silences SIX1 in NSCLC cells

**Figure 1:**
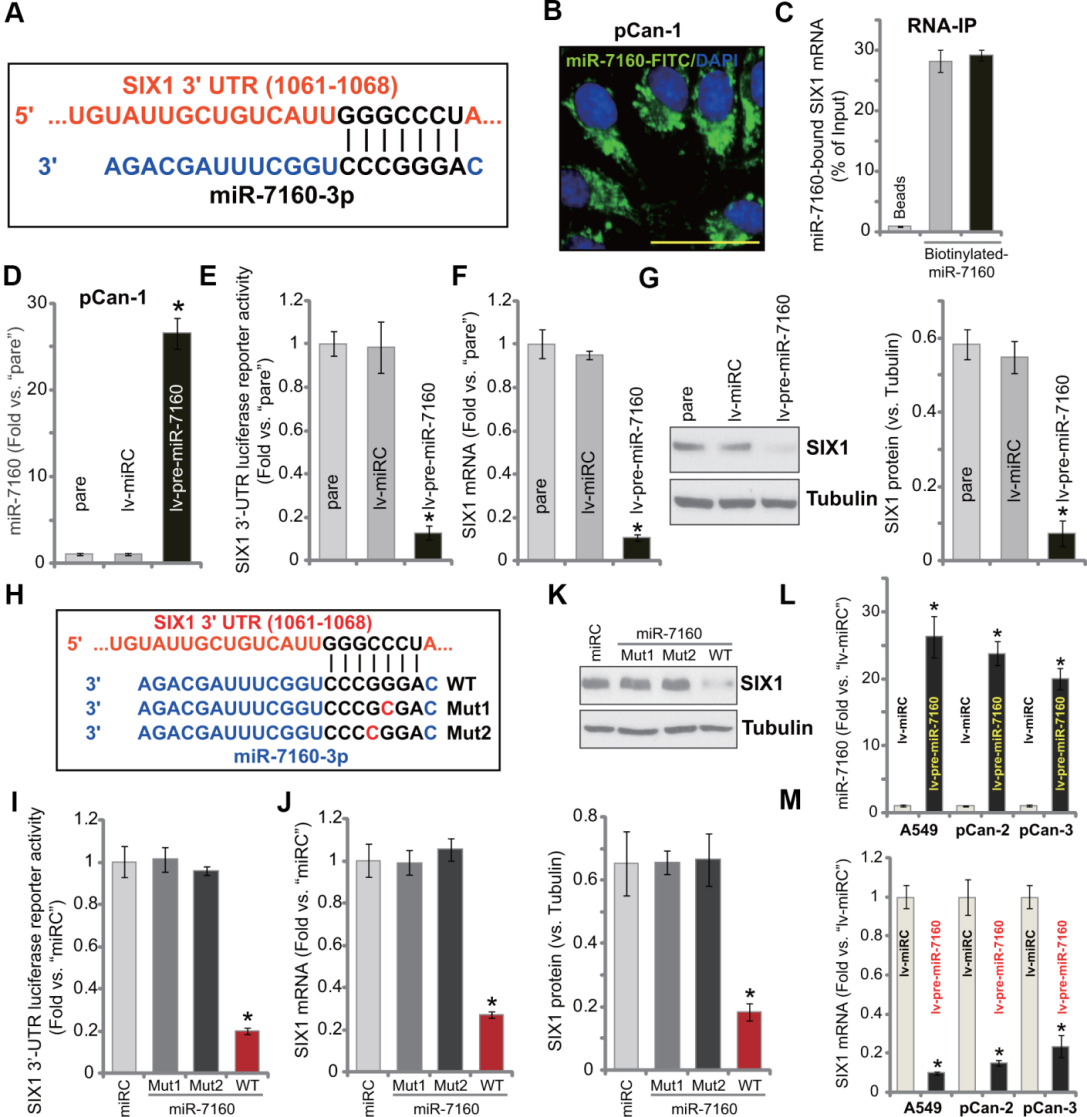
**miR-7160 binds and silences *SIX1* in NSCLC cells.** miR-7160 (-3p) putatively targets 3’-UTR of SIX1 (position of 1061-1068) (**A**). Immuno-fluorescence results showed that miR-7160 localized in the cytoplasm in pCan-1 NSCLC cells (**B**). Biotinylated-miR-7160 immunoprecipitated with *SIX1* mRNA in pCan-1 cells (**C**). Stable pCan-1 NSCLC cells, bearing a pre-miR-7160-expression lentiviral construct (“lv-pre-miR-7160”) or non-sense miRNA control lentiviral construct (“lv-miRC”), were established, expression of listed genes was tested by qPCR and Western blotting analyses (**D**, **F**, **G**), with SIX1 3’-UTR luciferase reporter activity tested as well (**E**). pCan-1 NSCLC cells were transfected with 500 nM of the wild-type (WT) or the mutant miR-7160 mimics (sequences listed in H), after 48h the *SIX1* 3’-UTR luciferase reporter activity (**I**) and its expression (**J**, **K**) were tested. A549 cells or primary NSCLC cells (pCan-2/pCan-3) were infected with lv-pre-miR-7160 or lv-miRC for 48h, expression of miR-7160 (**L**) and *SIX1* mRNA (**M**) was tested by qPCR. “pare” stands for the parental cells (same for all Figures). Data were presented as mean ± SD (n=5), and results normalized. *P< 0.05 vs. “lv-miRC”/“miRC” cells. Experiments in this figure were repeated five times with similar results obtained. Bar= 50 μm (**B**).

The miRNA database, TargetScan (V7.2), was consulted to search possible miRNAs binding the 3’-UTR of *SIX1*. The miRNAs were further verified by other miRNA databases (miRBase, miRNAmap, and miRTarbase). The bioinformatics analyses identified miR-7160 (-3p) that putatively targets the 3’-UTR of *SIX1* (position of 1061-1068) (Figure 1A). miR-7160 mainly localized in the cytoplasm of primary NSCLC cells (pCan-1) (Figure 1B). RNA immunoprecipitation (RNA-IP) experiments confirmed a direct binding between miR-7160 and *SIX1* mRNA (Figure 1C). *SIX1* mRNA immunoprecipitated with biotinylated-miR-7160 in pCan-1 cells (Figure 1C).

A lentiviral construct expressing pre-miR-7160 (lv-pre-miR-7160) was established. It was transduced to pCan-1 cells. With puromycin selection stable cells were established. The mature miR-7160 (-3p) expression increased over 25 folds in stable pCan-1 cells with lv-pre-miR-7160 (Figure 1D). Forced miR-7160 overexpression led to dramatic reduction of *SIX1* 3’-UTR luciferase reporter activity in pCan-1 cells (Figure 1E). *SIX1* mRNA (Figure 1F) and protein (Figure 1G) expression was significantly downregulated.

To further confirm a direct interaction between miR-7160 and *SIX1* mRNA, we created two mutant miR-7160 mimics (mutations at the binding site to *SIX1* 3’-UTR, Figure 1H). The two mutants, namely Mut1 and Mut2, as well as the wild-type (WT) miR-7160 mimics were individually transfected to pCan-1 cells. WT miR-7160 mimic potently decreased *SIX1* 3’-UTR luciferase reporter activity (Figure 1I) as well as *SIX1* mRNA (Figure 1J) and protein (Figure 1K) expression. The two mutants were, however, ineffective (Figure 1I–1K). In established A549 cells and primary NSCLC cells-derived from two other patients, pCan-2/pCan-3, lv-pre-miR-7160 led to significant increase of mature miR-7160 expression (Figure 1L). Consequently, *SIX1* mRNA expression was inhibited (Figure 1M). These results show that miR-7160 silenced *SIX1* in NSCLC cells.

### miR-7160 overexpression inhibits NSCLC cell growth, proliferation, migration and invasion

**Figure 2:**
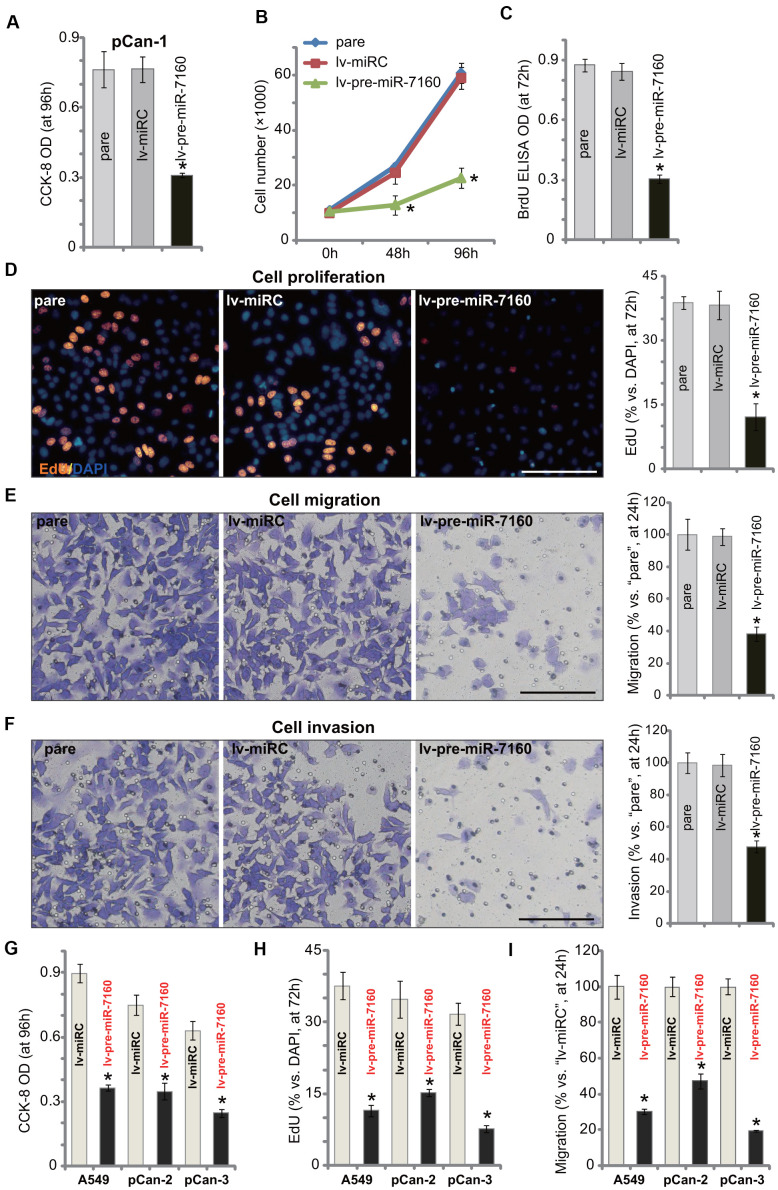
**miR-7160 overexpression inhibits NSCLC cell growth, proliferation, migration and invasion.** The primary NSCLC cells (pCan-1/pCan-2/pCan-3) or A549 cells, bearing the pre-miR-7160-expression lentiviral construct (“lv-pre-miR-7160”) or non-sense miRNA control lentiviral construct (“lv-miRC”), were cultured for applied time periods; Cellular functions, including viability (**A**, **G**), growth (**B**), proliferation (**C**, **D**, **H**), migration (**E**, **I**) and invasion (**F**) were tested by the assays mentioned in the text, with results quantified. Data were presented as mean ± SD (n=5), and results normalized. *P< 0.05 vs. “lv-miRC” cells. Experiments in this figure were repeated five times with similar results obtained. Bar= 100 μm (**D**–**F**).

SIX1 overexpression is important for proliferation, migration and invasion as well as EMT and chemo-resistance of NSCLC [25–28]. In Figure 1 we show that miR-7160 silenced SIX1, we tested its effect on functions of NSCLC cells. CCK-8 assay results demonstrated that forced overexpression of miR-7160, by lv-pre-miR-7160 (see Figure 1), largely inhibited viability of pCan-1 cells (Figure 2A). Figure 2B showed that lv-pre-miR-7160-expressing pCan-1 cells grew significantly slower than control cells with lv-miRC. In pCan-1 cells, miR-7160 overexpression inhibited BrdU incorporation (Figure 2C) and EdU-positive nuclei ratio (Figure 2D), indicating proliferation inhibition. In pCan-1 cells with lv-pre-miR-7160, migration and invasion were significantly inhibited (Figure 2E, 2F). The lv-miRC had no significant effect on the cellular functions of pCan-1 cells (Figure 2A–2F). In A549 cells and other primary NSCLC cells (pCan-2/pCan-3), lv-pre-miR-7160 similarly inhibited cell viability (CCK-8 OD, Figure 2G) and proliferation (EdU-positive nuclei ratio, Figure 2H). *In vitro* cell migration was inhibited as well (Figure 2I). Therefore, ectopic overexpression of miR-7160 inhibited NSCLC cell growth, proliferation, migration and invasion.

### miR-7160 overexpression provokes apoptosis in NSCLC cells

**Figure 3:**
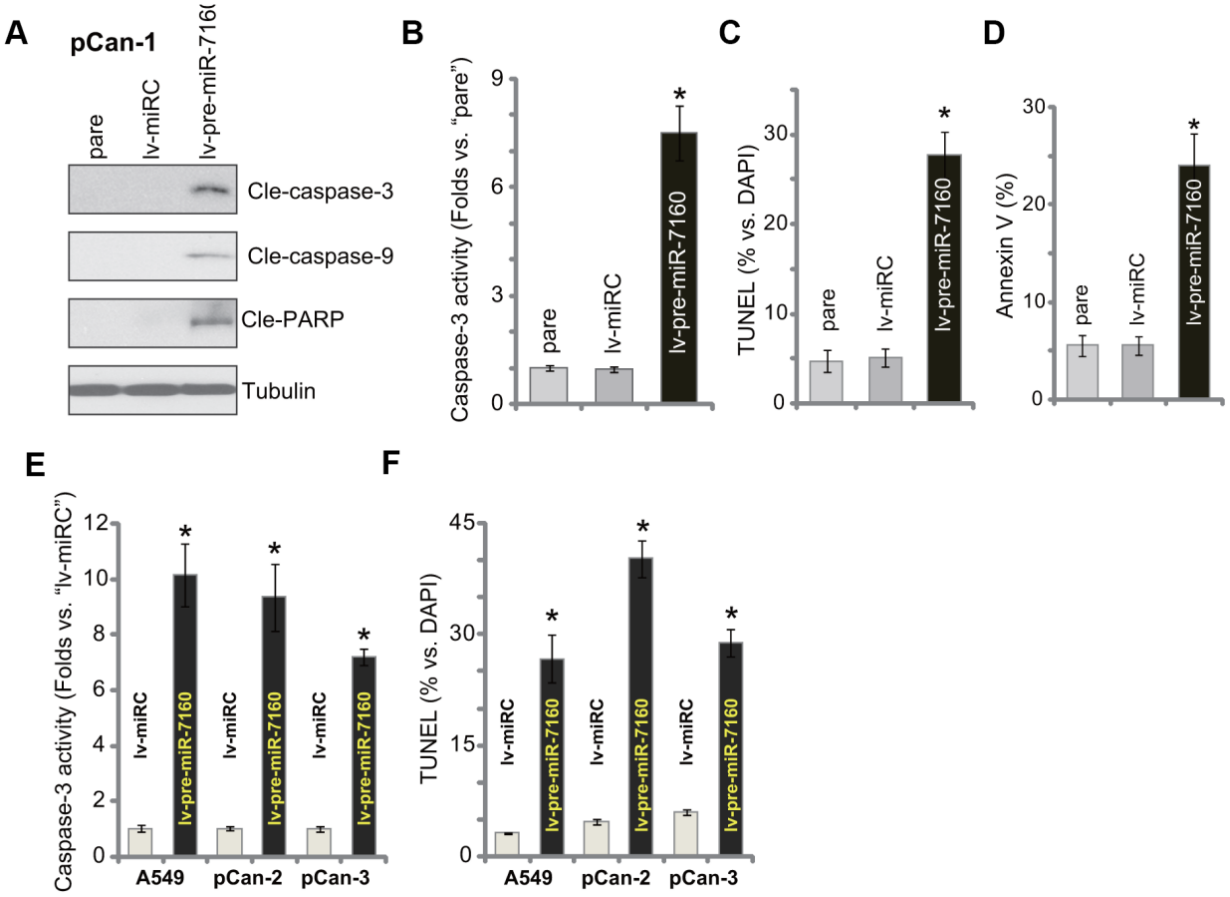
**miR-7160 overexpression provokes apoptosis in NSCLC cells.** The primary NSCLC cells (pCan-1/pCan-2/pCan-3) or A549 cells, bearing the pre-miR-7160-expression lentiviral construct (“lv-pre-miR-7160”) or non-sense miRNA control lentiviral construct (“lv-miRC”), were cultured for 48h; Caspase activation (**A**, **B**, **E**), and cell apoptosis (**C**, **D**, **F**) were tested by the assays mentioned in the text, with results quantified. Data were presented as mean ± SD (n=5), and results normalized. *P< 0.05 vs. “lv-miRC” cells. Experiments in this figure were repeated five times with similar results obtained.

We tested potential activity of miR-7160 on cell apoptosis. As demonstrated, in pCan-1 NSCLC cells-expressing lv-pre-miR-7160, we detected cleavages of caspase-3, caspase-9 and poly (ADP-ribose) polymerase (PARP) (Figure 3A). The caspase-3 activity increased over seven folds in miR-7160-overexpressed cells (Figure 3B). The TUNEL-positive cell nuclei percentage was significantly increased in lv-pre-miR-7160-expressing pCan-1 cells (Figure 3C), indicating apoptosis activation. It was further supported by the increased ratio of Annexin V-positive staining (Figure 3D). The control lv-miRC failed to induce apoptosis activation in pCan-1 cells (Figure 3A–3D). In established A549 cells and other primary NSCLC cells (pCan-2/pCan-3), the caspase-3 activity (Figure 3E) and TUNEL-positive nuclei ratio (Figure 3F) were significantly increased by lv-pre-miR-7160. Collectively, miR-7160 overexpression provoked apoptosis in NSCLC cells.

### miR-7160 inhibition promotes NSCLC cell progression

**Figure 4:**
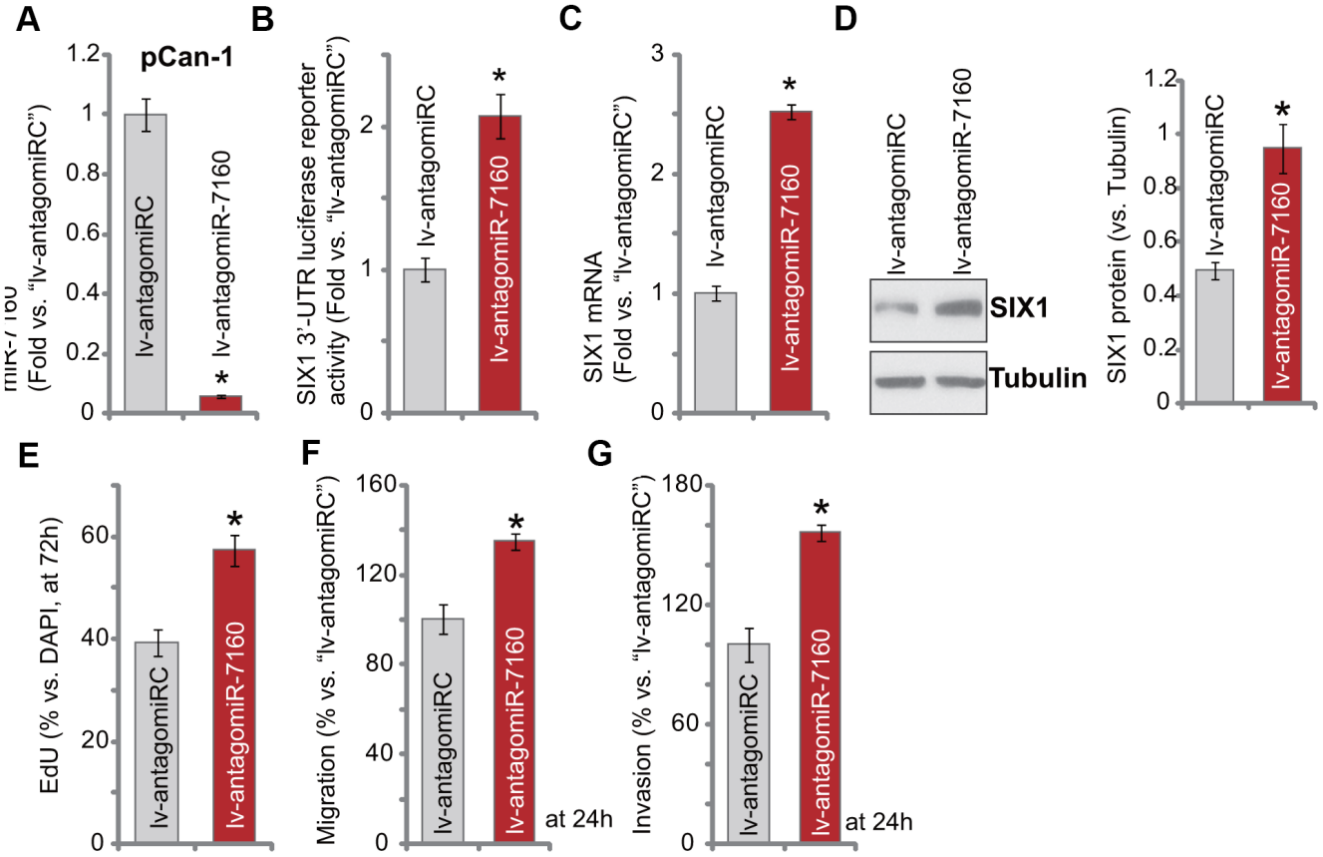
**miR-7160 inhibition promotes NSCLC cell progression.** The pCan-1 primary NSCLC cells, bearing the pre-miR-7160 anti-sense lentiviral construct (“lv-antagomiR-7160”) or non-sense anti-sense control lentiviral construct (“lv-antagomiRC”), were established, and cells cultured for applied time periods; Expression of listed genes was tested by qPCR and Western blotting analyses (**A**, **C**, **D**), with SIX1 3’-UTR luciferase reporter activity tested as well (**B**). Cellular functions, including cell proliferation (**E**), migration (**F**) and invasion (**G**) were tested by the assays mentioned in the text, with results quantified. Data were presented as mean ± SD (n=5), and results normalized. *P< 0.05 vs. “lv-antagomiRC” cells. Experiments in this figure were repeated five times with similar results obtained.

We further hypothesized that miR-7160 inhibition should increase SIX1 expression. A lentiviral construct containing pre-miR-7160 anti-sense sequence, lv-antagomiR-7160, was transduced to pCan-1 cells. Through puromycin selection stable cells were established. As compared to the control pCan-1 cells with non-sense construct (“lv-antagomiRC”), miR-7160 expression in lv-antagomiR-7160 cells decreased over 90% (Figure 4A). Conversely, lv-antagomiR-7160 resulted in over two-fold increase of *SIX1* 3’-UTR luciferase reporter activity (Figure 4B). Expression of *SIX1* mRNA (Figure 4C) and protein (Figure 4D) was subsequently elevated. Functional studies demonstrated lv-antagomiR-7160 augmented pCan-1 cell proliferation (nuclear EdU ratio increase, Figure 4E), migration and invasion (“Transwell” assays, results quantified in Figure 4F, 4G). These results show that miR-7160 inhibition augmented SIX1 expression and NSCLC cell progression *in vitro*.

### Altering miR-7160 expression is ineffective on the function of SIX1 KO NSCLC cells

**Figure 5:**
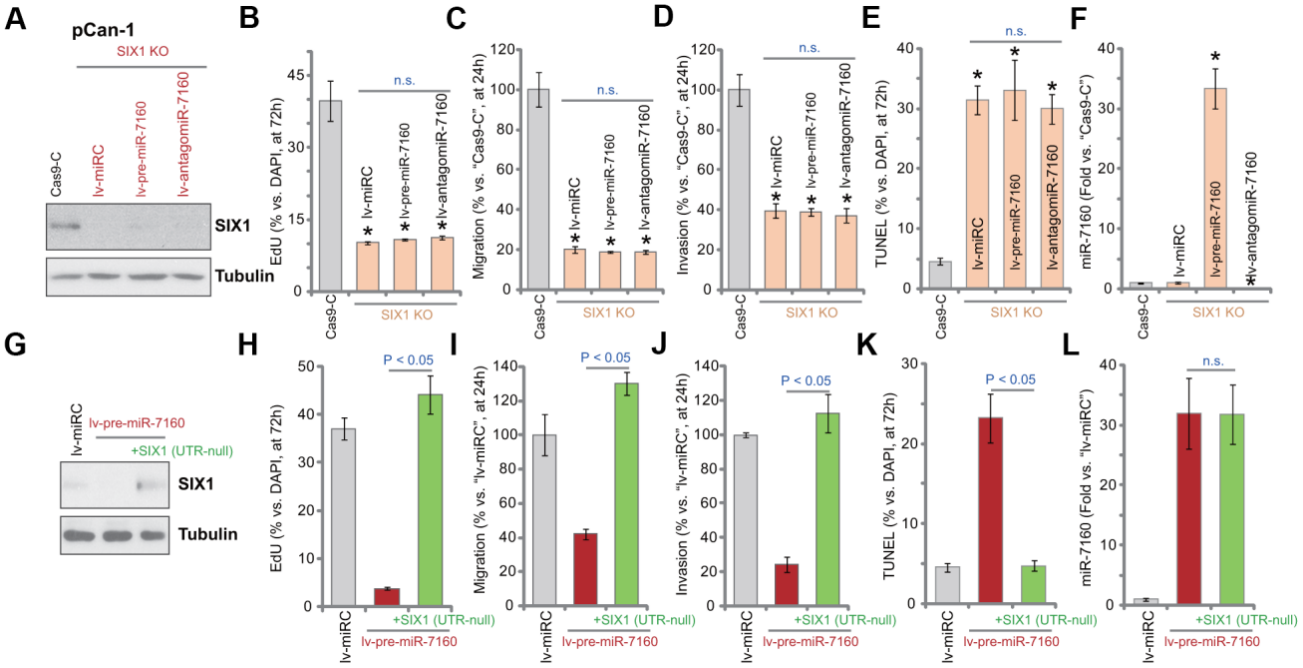
**Altering miR-7160 expression is ineffective on the function of SIX1 KO NSCLC cells.** The pCan-1 NSCLC cells with the CRISPR/Cas9-SIX1-KO-GFP construct (SIX1 KO cells) were further infected with pre-miR-7160-expression lentivirus (lv-pre-miR-7160), pre-miR-7160 anti-sense lentivirus (lv-antagomiR-7160) or the control non-sense miRNA lentivirus (lv-miRC), control cells were transduced with CRISPR/Cas9 empty construct (Cas9-C), stable cells were established; Expression of listed proteins was tested by Western blotting assays (**A**); Cells were further cultured for applied time periods, cellular functions, including cell proliferation (nuclear EdU ratio, **B**), migration and invasion (“Transwell” assays, **C**, **D**), as well as cell apoptosis (nuclear TUNEL staining, **E**), were tested, with results quantified; Expression of miR-7160 was tested by qPCR (**F**). The pCan-1 cells bearing the pre-miR-7160-expression lentiviral construct (“lv-pre-miR-7160”) were transfected with or without a lentiviral 3’-UTR-null SIX1 expression construct: +SIX1 (UTR-null); Control cells were transfected with the control non-sense miRNA lentivirus (lv-miRC); Expression of listed proteins was tested by Western blotting (**G**); Cells were further cultured for applied time periods, cell proliferation (**H**), migration and invasion (**I**, **J**), as well as cell apoptosis (**K**) were tested, with results quantified; Expression of miR-7160 was tested by qPCR (**L**). Data were presented as mean ± SD (n=5), and results normalized. *P< 0.05 vs. “Cas9-C” cells (**B**–**F**). n.s. stands for no statistic difference (**B**–**E**, **L**). Experiments in this figure were repeated five times with similar results obtained.

A CRISPR/Cas9-SIX1-KO-GFP construct was transduced to pCan-1 cells. FACS-mediated GFP sorting and puromycin selection were then carried out to select stable cells: SIX1 KO cells. Western blotting assay results, Figure 5A, confirmed that SIX1 protein was depleted in the SIX1 KO cells. SIX1 KO largely inhibited pCan-1 cell proliferation (nuclear BrdU incorporation, Figure 5B), migration and invasion (“Transwell” assays, Figure 5C, 5D), while inducing cell apoptosis (TUNEL-positive nuclei ratio increase, Figure 5E). Thus, mimicking lv-pre-miR-7160, SIX1 KO produced significant anti-NSCLC cell activity. Importantly, in SIX1 KO cells, exogenously altering miR-7160 expression by lv-pre-miR-7160 or lv-antagomiR-7160 (Figure 5F) failed to change cellular functions (Figure 5B–5E). Therefore, in SIX1 KO cells miR-7160-induced anti-cancer activity was compromised.

If miR-7160-induced anti-NSCLC cell activity was due to silencing SIX1, restoring SIX1 expression should abolish miR-7160-induced actions. A lentiviral 3’-UTR-null SIX1 expression construct, SIX1 (UTR-null), was transduced to pCan-1 cells. As shown SIX1 (UTR-null) restored SIX1 protein expression in lv-pre-miR-7160-expressed pCan-1 cells (Figure 5G). Functional studies demonstrated that SIX1 (UTR-null) completely reversed lv-pre-miR-7160-induced inhibition on pCan-1 cell proliferation (Figure 5H), migration (Figure 5I) and invasion (Figure 5J), and blocking cell apoptosis (Figure 5K). Lv-pre-miR-7160-induced miR-7160 overexpression was unchanged by SIX1 (UTR-null) (Figure 5L). These results further supported that miR-7160-induced anti-NSCLC cell activity is through SIX1 silencing.

### Forced miR-7160 overexpression silences SIX1 and inhibits NSCLC xenograft growth in mice

**Figure 6:**
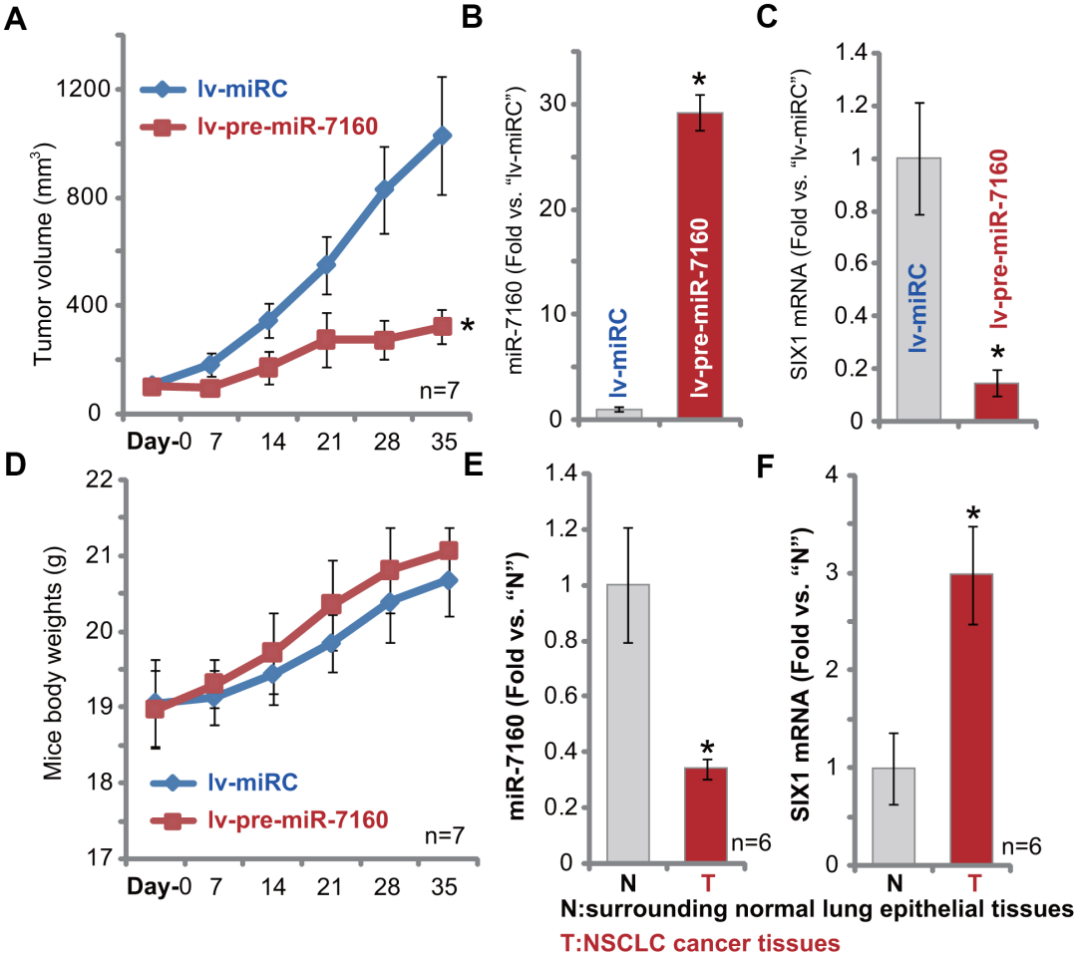
**Forced miR-7160 overexpression silences SIX1 and inhibits NSCLC xenograft growth in mice.** The pCan-1 NSCLC cells were inoculated thorough *s.c.* injection to SCID mice (n=7 per group). Within three weeks tumor xenografts were established (Day-0), with tumor volume around 100 mm^3^. NSCLC xenografts were intratumorally injected with either lv-pre-miR-7160 or lv-miRC. Tumor volumes (**A**) and mouse body weights (**D**) were recorded every 7 days. At Day-7, one NSCLC tumor xenograft per group was isolated, each tumor was randomly cut into five small pieces (n=5, for **B**, **C**), and expression miR-7160 (**B**) and *SIX1* mRNA (**C**) in tumor lysates tested by qPCR. The relative expression miR-7160 (**E**) and *SIX1* mRNA (**F**) in NSCLC cancer tissues (“T”) and paired surrounding normal lung epithelial tissues (“N”) was shown. Data were presented as mean ± SD, and results normalized. * *p*< 0.05 *vs.* lv-miRC control tumors (**A**–**C**) or “N” tissues (**D**, **E**).

In order to study the potential effect of miR-7160 on NSCLC cell growth *in vivo*, we injected pCan-1 cells to the flanks of SCID mice. NSCLC xenografts were established within three weeks, when tumor volumes reached approximately 100 mm^3^ (“Day-0”). NSCLC xenografts were than subjected to intratumoral administration of either lv-pre-miR-7160 or lv-miRC. Tumor growth curve results, Figure 6A, demonstrated that NSCLC xenografts with lv-pre-miR-7160 injection grew significantly slower than control NSCLC xenografts with lv-miRC. Tumor volumes in lv-pre-miR-7160 group were significantly lower than lv-miRC tumors (Figure 6A). On Day-7, one tumor from each group was individually isolated. The qPCR assay results showed that mature miR-7160 levels increased over 25-30 folds in lv-pre-miR-7160-injected NSCLC xenografts (Figure 6B), with *SIX1* mRNA significantly downregulated (Figure 6C). The animal body weights were not significantly different between the two groups (Figure 6D). Neither did we notice apparent signs of toxicity. Therefore, forced miR-7160 overexpression silenced SIX1 and inhibited NSCLC xenograft growth in SCID mice.

### miR-7160 is downregulated in human NSCLC tissues

At last we tested expression of miR-7160 in human NSCLC tissues. A total of six (6) NSCLC cancer tissues from primary NSCLC patients [29] were tested. As demonstrated, miR-7160 expression in NSCLC cancer tissues (“T”) was significantly lower than that in the paired surrounding normal lung epithelial tissues (“N”) (Figure 6E). It correlated with *SIX1* mRNA upregulation in cancer tissues (Figure 6F). Therefore, miR-7160 is downregulated in human NSCLC tissues and SIX1 is upregulated.

## DISCUSSION

miRNAs are commonly dysregulated in NSCLC and other lung cancers, which are extremely important for cancer initiation, tumorigenesis, and progression as well as treatment resistance, and prognosis determination [23, 24]. Expression and potential functions of miR-7160 in human cancer have not been studied thus far.

Our results here suggest that miR-7160 should be a tumor suppressor in NSCLC. In A549 and primary NSCLC cells, forced overexpression of miR-7160 potently inhibited cell growth, proliferation, migration and invasion. It also provoked cell apoptosis. Conversely, miR-7160 inhibition, by lv-antagomiR-7160, promoted NSCLC cell proliferation, migration and invasion. Intratumoral administration of lv-pre-miR-7160 potently inhibited NSCLC xenograft growth in SCID mice. Importantly, levels of miR-7160 are downregulated in human NSCLC tissues. Therefore, miR-7160 exerted tumor-suppressive activity in NSCLC.

miRNA-induced SIX1 silencing has proven to be a good strategy to inhibit NSCLC cells. Xia et al., reported that miR-204 silenced SIX1 and inhibited NSCLC cell invasion, proliferation and EMT [28]. Liu et al., found that miR-186-5p targeted SIX1 and inhibited cisplatin resistance in NSCLC cells [25]. Ge et al., have demonstrated that lncRNA FOXD2-AS1 sponged miR-186-5p to increase SIX1 expression, conferring cisplatin resistance in NSCLC cells [26].

Our results showed that miR-7160 targeted and silenced SIX1 in NSCLC cells. RNA-IP experiments confirmed a direct binding between miR-7160 and *SIX1* mRNA in NSCLC cells. In NSCLC cells forced overexpression of miR-7160 potently inhibited *SIX1* 3’-UTR luciferase reporter activity and its expression. Conversely, SIX1 expression was increased with miR-7160 inhibition by lv-antagomiR-7160. Importantly, miR-7160 mimics with mutations at the binding sites to *SIX1* were unable to alter *SIX1* 3’-UTR luciferase reporter activity and its expression. *In vivo* lv-pre-miR-7160 intratumoral administration downregulated *SIX1* mRNA in NSCLC xenografts. Importantly, in human NSCLC tissues miR-7160 expression is downregulated and correlated with *SIX1* mRNA upregulation. Thus, miR-7160 is a *SIX1*-targeting miRNA in NSCLC.

We here provided strong evidence to support that miR-7160-induced anti-NSCLC activity is due to silencing *SIX1*. Restoring SIX1 expression, by an UTR-null SIX1 construct, reversed lv-pre-miR-7160-induced anti-NSCLC cell activity. CRISPR/Cas9-inudced SIX1 knockout mimicked miR-7160-induced actions. Importantly, miR-7160-induced anti-cancer activity was compromised in SIX1-KO NSCLC cells. In other words, miR-7160 was completely ineffective in SIX1-KO NSCLC cells. These results suggest that SIX1 silencing should be the primary reason of miR-7160-induced anti-NSCLC cell activity.

The current clinical treatment options for NSCLC, including tumor resection, platinum-based chemotherapies, radiation, and newly-developed molecularly-targeted therapies, are not satisfactory [2, 4]. There is an urgent need to explore novel and more efficient anti-NSCLC strategies. The results of this study show that miR-7160, a potential tumor suppressor, can inhibit NSCLC cell growth by silencing its target gene SIX1. It may represent a promising therapeutic strategy and diagnosis marker for NSCLC.

## MATERIALS AND METHODS

### Chemicals and reagents

Antibodies were provided by Abcam (Cambridge, MA) and Cell Signaling Tech (Shanghai, China). Fetal bovine serum (FBS) and all other cell culture reagents were purchased from Hyclone (Logan, UT). Puromycin, polybrene, cell-counting kit 8 (CCK-8), and other chemicals were obtained from Sigma-Aldrich Chemicals Co. (St. Louis, Mo). Primers and viral constructs were designed and verified by Genechem Co. (Shanghai, China) unless otherwise specified. Annexin V, propidium iodide (PI), TUNEL dye, and reagents for PCR and transfection assays were obtained from Thermo-Fisher Invitrogen (Shanghai, China).

### Cell culture

A549 cell line was provided by Dr. Chen [30] and cultured as described [30]. The primary human NSCLC cells, provided by Dr. Jiang [31, 32], were derived from three NSCLC patients, namely pCan-1, pCan-2, pCan-3. The primary cells were cultured as previously described [31, 32]. For the established and primary human cells short tandem repeat (STR) profiling, population doubling time, and morphology were routinely checked to verify the genotypes. The protocols for using human cells were approved by the Ethics Committee of Zhengzhou University in accordance to Declaration of Helsinki.

### Quantitative real-time PCR (qPCR)

Total RNA was extracted by TRIzol reagents [33], reversely transcripted to complementary DNA (cDNA). qPCR analyses were carried out using a SYBR Premix Ex Taq™ kit [30] under the ABI Prism 7500 Fast Real-Time PCR system (Applied Biosystems). *GAPDH* was always tested as the reference gene and internal control. A 2^−ΔΔ*C*t^ method was utilized to quantify targeted mRNAs. miR-7160 expression was examined by a TaqMan microRNA qPCR assay kit, and *U6* tested as the internal control. All primers are listed in Table 1.

**Table 1:** Sequences utilized in this study.

**Gene name**	**Forward primer (5’-3’)**	**Reverse primer (5’-3’)**
*miR-7160*	TGCTGAGGTCCGGGCTGT	GAACATGTCTGCGTATCTC
*U6*	CTCGCTTCGGCAGCACAT	TTTGCGTGTCATCCTTGCG
*GAPDH*	GTCTCCTCTGACTTCAACAGCG	ACCACCCTGTTGCTGTAGCCAA
*SIX1*	AAGGAGAAGTCGAGGGGTGT	TGCTTGTTGGAGGAGGAGTT
		
SIX1 KO	Target DNA Sequence	PAM Sequence
SIX1 sgRNA	GCCGTCGTTTGGCTTTACGC	AGG

### SIX1 UTR luciferase reporter assay

As reported elsewhere [34], *SIX1* 3′-UTR sequence containing the predicted miR-7160 binding sites, position 1061-1068, was amplified by Genechem and sub-cloned into a pMIR-REPORT miRNA expression reporter vector (provided by Dr. Chen [30]), thus generating a luciferase reporter plasmid pMIR-SIX1-3′-UTR. The latter was transfected to NSCLC cells, which were subjected to genetic modifications. The 3′-UTR luciferase reporter activity was detected under a dual-luciferase reporter assay system (Promega, Shanghai, China).

### Forced miR-7160 overexpression or inhibition

The miR-7160 precursor sequence (“pre-miR-7160”, UGCUGAGGUCCGGGCUGUGCCCCGUACCGGACAGGGCCCUGGCUUUAGCAGA) and the corresponding anti-sense sequence were synthesized by Genechem (Shanghai, China), which were individually sub-cloned into a GV369 lentiviral construct (from Dr. Chen [30]). The construct, as well as the lentivirus-packing helper plasmids, were co-transfected to HEK-293T cells, generating pre-miR-7160-expressing lentivirus (lv-pre-miR-7160) and pre-miR-7160 anti-sense-expressing lentivirus (lv-antagomiR-7160). The lentivirus was added to NSCLC cells (cultured in polybrene-containing complete medium). Following selection by puromycin (5.0 μg/ml, for 4-5 passages), stable cells were established where expression of mature miR-7160 (sequence: UGCUGAGGUCCGGGCUGUGCC) was tested by qPCR.

### Transfection of miR-7160 mimic

At 50-60% of confluence NSCLC cells were seeded into six-well plates. Lipofectamine 2000 reagent was utilized to transfect the wild-type (“WT”) or the mutant (“Mut”) miR-7160 mimics (each at 500 nM, synthesized by Genechem). Transfection lasted for 48h.

### RNA immunoprecipitation (RNA-IP)

NSCLC cells, transfected with a biotinylated-miR-7160 mimic (wild-type or mutants), were homogenized by cell lysis buffer. RNA-IP was carried out by adding the streptavidin-coated magnetic beads into the cell lysates [35]. The biotinylated-miR-7160-bound beads was purified [30], and *SIX1* mRNA expression tested by qPCR. Its expression was always normalized to “Input” controls.

### Cell viability

At 3000 cells per well, NSCLC cells were seeded into 96-well tissue-culture plates and cultured for 96h. Ten μL of CCK-8 reagent was added for another two hours, and CCK-8 absorbance tested at 450 nm.

### Transwell migration/invasion assay

NSCLC cells (5×10^4^ cells per chamber, cultured into serum-free medium) were added to the upper surface of Transwell chambers (BD Biosciences, Shanghai, China). The lower chambers were filled with complete medium (containing 12% FBS). Cells were allowed to migrate for 24h. Migrated cells in the lower chambers were fixed, stained, and counted. For invasion assays, Matrigel (Sigma) was added to chamber surfaces.

### EdU assay

NSCLC cells (at 1×10^5^ cells per well) were seeded into six-well plates and cultured for 72h. An 5-ethynyl-20-deoxyuridine(EdU) Apollo-567 kit (RiboBio, Guangzhou, China) was utilized to test and quantify cell proliferation, with EdU ratio (EdU/DAPI×100%) determined from at least 500 nuclei in five random views (1×100) of each condition.

### BrdU ELISA

As described [36], NSCLC cells with applied genetic modifications were subjected to BrdU incorporation testing, using a BrdU ELISA kit (Roche Diagnostics Basel, Switzerland) according to the manufacturer’s protocol. The BrdU ELISA absorbance at 405 nm was recorded.

**Cell apoptosis assays**

The detailed protocols of routine apoptosis assays, including Annexin V fluorescence activated cell sorting (FACS) and nuclear TUNEL staining, were described in detail in other studies [37, 38].

### Western blotting

In brief, quantified protein lysates (30 μg per treatment) were separated by 10-12% SDS-PAGE gels [39] and transferred to polyvinylidene difluoride (PVDF) blots (Merck-Millipore). The resulting blots were blocked and incubated with the indicated primary and, subsequently, secondary antibodies. Binding of antibody-antigen was examined and visualized by an enhanced chemiluminescence (ECL) detection kit (Roche, Shanghai, China).

### SIX1 knockout

The lenti-CRISPR/Cas9-SIX1-KO-GFP plasmid was constructed by Genechem. The sgRNA sequence was listed in Table 1. NSCLC cells were initially plated into six-well plates (at 8 ×10^4^ cells per well) and transfected with the construct. GFP-positive cells were sorted by FACS and the monoclonal NSCLC cells distributed to 96-well plates. Cells were further cultured in puromycin-containing complete medium for four passages to establish stable cells, where *SIX1* knockout (KO) was screened by qPCR and Western blotting assays.

### UTR-null SIX1

The UTR-null SIX1 cDNA, constructed by Genechem, was sub-cloned into the GV369 lentiviral vector. The vector together with the lentivirus-packing helper plasmids were co-transfected into HEK-293T cells, generating lentivirus. UTR-null SIX1-expressing lentivirus was added to NSCLC cells (cultured in polybrene-containing complete medium). Following selection by puromycin, stable cells were established.

### Human tissues

NSCLC cancer tissues and paired surrounding lung epithelial tissues, from six primary NSCLC patients (male, 46 to 64-year old, stage-III), were provided by Dr. Li at Wenzhou Medical University [29]. The protocols were approved by the Ethics Committee of Zhengzhou University, in accordance to Declaration of Helsinki.

### *In vivo* tumor growth assay

The pCan-1 NSCLC cells (1×10^7^ cells per mouse, cells in Matrigel-containing serum-free medium) were injected subcutaneously (*s.c.*) into the flanks of severe combined immunodeficient mice (SCID) mice. Mice weighted 18.5-19.2g and were from Soochow University Animal facility (Suzhou, China). When the volume for each tumor reached approximately 100 mm^3^ (“Day-0”), mice were randomly assigned into two groups. Group I received intratumoral administration of lv-pre-miR-7160. Group II received lv-miRC administration. Tumor volumes were recorded under a previously described formula [40]. All animal procedures were approved by the Experimental Animal Ethical Committee of Zhengzhou University, in accordance to Declaration of Helsinki.

### Statistical analysis

Data were normally distributed and presented as means ± standard deviations (SD). One-way ANOVA and Student-Newman-Keuls post hoc test were performed to determine statistically differences among multiple groups (SPSS 23.0, SPSS Co. Chicago, IL). When comparing the difference between two specific groups, a two-tailed Student’s *t*-test (Excel 2007, Microsoft) was utilized. *P*<0.05 was considered statistically different.
